# Characteristics and occupational risk assessment of occupational silica-dust and noise exposure in ferrous metal foundries in Ningbo, China

**DOI:** 10.3389/fpubh.2023.1049111

**Published:** 2023-02-09

**Authors:** Donghui Duan, Pengbo Leng, Xiaohai Li, Guochuan Mao, Aihong Wang, Dandan Zhang

**Affiliations:** Ningbo Municipal Center for Disease Control and Prevention, Ningbo, China

**Keywords:** risk assessment, occupational and environmental exposure, ferrous metal, silica-dust exposure, noise exposure

## Abstract

**Introduction:**

To investigate the major existing occupational hazards and to assess the occupational health risks for ferrous metal foundries (FMFs) in Ningbo, China.

**Methods:**

Unified questionnaires were formulated to investigate the information on the basic situations, occupational hazards, and occupational health management for 193 FMFs in Ningbo. Furthermore, we used the semi-quantitative risk assessment model, which was developed by the International Council on Mining and Metals (ICMM), to assess occupational health risks for 59 of 193 the FMFs.

**Results:**

The casting process of FMFs in Ningbo was mainly divided into sand casting and investment casting, and silica-dust and noise were the major occupational hazards in both sand casting and investment casting foundries. Silica-dust mainly occurred in industries with such work as sand handling, modeling, falling sand, and sand cleaning, with the median of the permissible concentration-time weighted average (PC-TWA) was 0.80, 1.15, 3.52, 0.83 mg/m^3^, respectively. The noise mainly existed in industries with such work as sand handling, core making, falling sand, sand cleaning, cutting and grinding, and smelting with median of PC-TWA was 81.72 dB(A), 82.93 dB(A), 90.75 dB(A), 80.18 dB(A), 90.05 dB(A), 82.70 dB(A), respectively. In addition, the results of the ICMM assessment model indicated that 100 and 98.7% of the jobs exposed to silica-dust and noise in 59 FMFs have an “intolerable risk” level of risks of causing pneumoconiosis and noise deaf, respectively.

**Discussion:**

The hazard risk of silica-dust and noise is serious for FMFs in Ningbo. It is necessary to supervise enterprises to improve operating environmental conditions, accelerate the reduction of silica-dust and noise exposure risks, and promote the healthy and sustainable development of the foundry industry.

## Introduction

As the largest developing country, China is experiencing one of the worst occupational health problems in the world and faced with more severe threats and challenges induced by occupational hazardous factors than most other countries. Over 200 million workers from at least 20 million enterprises are at risk of occupational diseases in China (1). It is estimated that at least one million subjects suffered from occupational diseases (OD) with nearly 30,000 newly diagnosed cases per year over the past decade, leading to a considerable burden on the society (2). The increase in OD incidence in China, in large part, is ascribed to serious occupational hazards in workplace, such as silica-dust and noise, and inadequate personal protective equipment (3).

Ningbo is an economic center of Zhejiang Province, which is also a coastal city with a population over than 9 million, with a high level of economic development comparing to the general situation of China (GDP per capita in 2021 $2,3846 vs.$1,2462) (4). With the rapid growth of social economy, the foundry industry has developed rapidly and has become a pillar industry in Ningbo (5). However, FMFs are one of the industries with serious dust hazards, and the newly diagnosed cases of pneumoconiosis in Ningbo in recent years were concentrated in this industry (6). Currently, 20% of newly diagnosed occupational hearing loss was observed in this industry, according to the investigation by Zhejiang Provincial Center for Disease Control and Prevention (7). Due to regulatory and legal measures, more and more companies and organizations give attention to employee health and safety, try to control occupational hazards, and seek to improve the level of worker safety (8, 9).

Occupational Health and Safety (OHS), defined as the science of the anticipation, recognition, evaluation and control of hazards arising in or from the workplace that could impair the health and wellbeing of workers, is an important issue for both employees and employers (10–12). As one critical process in OHS practice, occupational health risk assessment (OHRA) is an effective tool to assess the risk of occupational hazards in workplace and take required control measures for providing safety (13, 14). Developed countries and international organizations have developed several OHRA methods, including the models from the United States Environmental Protection Agency (EPA) (15), the United Kingdom's Control of Substances Hazardous to Health Essentials (COSHH Essentials) (16), Australia (17), Romania (18), the International Council on Mining and Metals (ICMM) (19), and Singapore (20). The ICMM model was used to evaluate the occupational health risk in this study mainly attributing to several reasons: first, the model has a broad scope of evaluated substances; second, the model could be applied to various industries, including ferrous metal casting industry; third, the model was based on qualitative or subjective descriptions, and less detailed information was required for use (13).

According to Ningbo Municipal Statistics Bureau, there were ~267 FMFs with over workers until June 2020 (5). It is necessary to understand the characteristics of occupational hazards in the main positions of the FMFs and evaluate the corresponding occupational hazard risks to provide a scientific basis for formulating policies of occupational disease prevention and control.

The primary purpose of this study was to investigate the exposure characteristics and occupational health risks of silica-dust and noise, and provide a basis for developing reasonable control measures to reduce the health risks for workers. Accordingly, the following two step were conducted successively: (1) occupational hygiene survey and field investigation for normally operating ferrous metal foundries (FMFs); (2) assess occupational health risk by using the model developed by ICMM.

## Methods

### Description of Ferrous metal casting industry

Ferrous metal casting industry, one of the most important foundry industries in Ningbo, is the process of smelting iron and steel metal into a liquid that meets certain requirements and pouring it into a mold, in order to obtain castings of predetermined shape, size, and properties, after cooling, solidification, and cleaning (21). Currently, commonly used process flow of casting including sand casting, investment casting, pressure casting and centrifugal casting, etc., (21). The process flow of sanding casting and investment casting were selected for this study. The inherent risk (IR) of ferrous metal casting industry was directly obtained from a normative document, Catalog of Classification and Management of Occupational Disease Hazard Risks in Construction Projects, formulated by the National Health Commission of China (22). Based on the document, the occupational hazards are assigned a level of risk according to the advice and consultation of China's top occupational health experts. Accordingly, the IR level of ferrous metal casting industry was classified as “severe” in this study. In addition, the enterprises classification standard was formulated by the National Bureau of Statistics of China (23).

### Occupational hygiene survey

Questionnaires were used to investigate the basic information of FMFs, including enterprise size, production process, job setting, and etc. For occupational health management information, engineering protection facilities, personal protective equipment and occupational hygiene management system formulation and implementation, and etc. were investigated. In order to maintain the quality of the survey, we formulated unified questionnaire, compiled survey operation technical manuals, and conducted technical training for investigators. In this study, the field measurements and interviews were performed by experts who had title of associate chief physician or senior engineer.

### Identification of occupational hazardous factors

Occupational hazardous factors were determined through field investigation, air sampling, and laboratory tests based on two occupational health standards in China, that is, the “Specifications of air sampling for hazardous substances monitoring in the workplace (GBZ 159)” and “Determination of toxic substances in workplace air (GBZ/T 160 and 300).” The levels of occupational hazards in FMFs were qualified by using the Chinese Occupational Exposure limits for Hazardous Agents in Workplace (GBZ 2.1-2019). Onsite measurement of noise was conducted according to the standard “The physical factor measurement in the workplace (GBZ/T189.8-2007).” The exposure levels of silica-dust and noise at various locations in the sand conditioning, molding and core making, melting, shakeout sand, shot blasting, and cutting and polishing had different degrees of exceed the permissible concentration time weighted average (PC-TWA) permitted by China. The evaluation of silica-dust and noise was based on the PC-TWA. “Qualified” or “Disqualified” was equal to the exposure level of risk factor not exceed the standard of PC-TWA or exceed the standard of PC-TWA, respectively. For silica-dust, the PC-TWA by China was 0.3 mg/m^3^ for free SiO_2_ content higher than 50% and lower than 80%, according to GBZ 2.1-2019. The permissible level of noise was 85 dB(A), according to GBZ 189.8-2007.

### Methodology for the ICMM model

The ICMM model was based on two factors: the inherent harmful consequences and their probability of occurrence, which were evaluated by four procedures step by step: hazard identification, hazard characterization, exposure assessment, and risk characterization. The detailed principles of the ICMM model were reported in previous publication (19). The ICMM model applies a matrix method to evaluate risk levels, including matrix combinations of health hazards and the probability of exposure occurring in a similar exposure group or process, as well as matrix combinations of health hazards and exposure levels with existing control measures.


$$\begin{array}{l} \text{RR}=\text{C}\times \text{PrE}\times \text{PeE}\times \text{U} \end{array}$$

In this equation, RR is the risk ratio; C is level of occupational hazard health consequences; PrE is the probability of exposure, based on the ratio of the exposure level (E) and occupational exposure limit (OEL); PeE is the length of exposure; U is uncertainty factor. The result of ICMM model (RR) was also converted into a classification of five risk levels, which could vary from 1 to 5: Level 1, RR < 20 represents a tolerable risk; Level 2, RR ranges from 20 to 70, which represents a potential risk; Level 3, RR ranges from 70 to 200, which represents a high risk; Level 4, RR ranges from 200 to 400, which represents a very high risk; Level 5, RR greater or equal to 400 represents an intolerable risk. Risk scoring for risk criteria is showed in Table 1. In this study, quantitative risk assessment was performed by statisticians who had background of medical or public health, and examined by experts subsequently.

**Table 1:** Risk scoring table for risk criteria.

**Risk criteria**	**Description**	**Score**
PrE^a^	Exposure rate lower than 50% of OEL	3
	50–100% of OEL^e^	6
	Above OEL	10
PeE^b^	One a year	0.5
	Short periods several times a month	2
	2–8 h on average during shift work.	6
	Over 8 h of exposure (within overtime and shiftwork)	10
C^c^	Exposure at this level does not harm the personnel	1
	Health effects are reversible and not a threat to one's life	15
	Undesirable health effects that are permanent or temporary but have little effect on one's quality of life and life expectancy	50
	Health effects that are usually permanent and can significantly decrease quality of life or life expectancy	100
RR^d^	Tolerable risk	< 20 (Level 1)
	Potential risk	20–70 (Level 2)
	High risk	70–200 (Level 3)
	Very high risk	200–400 (Level 4)
	Intolerable risk	≥400 (Level 5)

a PrE, Probability of exposure criteria;

b PeE, Duration of Exposure Criteria;

c C, Severity of Consequences Criteria;

d RR, Result of risk assessment;

e OEL, occupational exposure limit.

Briefly, the process of performing the ICMM model had two phases in the current study. The first phase was identification of risk criteria. According to previous investigations and publications, three risk criteria were considered for this method, which included the probability of exposure to hazardous factors (PrE), the duration of exposure criteria (PeE), and the severity of consequence (C).

The second phase was determining the risk scoring system and risk level. To rate this criterion for PrE, control measures for any potential hazard are required to be assessed directly or indirectly. In direct assessments, the exposure level needs to be measured and compared with the standards. In indirect assessments, documents of recent measurements can be used. If the exposure rate was lower than 50% of occupational exposure limit (OEL), the corresponding score was three points. If the exposure rate was between 50 and 100% of OEL, the corresponding score was 6 points. While if the exposure rate was over the OEL, the corresponding score was 10 points. For the duration of exposure criteria, the exposure duration criterion was set at 4 levels: one a year, short periods several times a month, 2–8 h on average during shift work, and over 8 h of exposure (within overtime and shiftwork), which were equal to scores of 0.5, 2, 6, 10 points, respectively. For severity of consequence, the score of 4 levels was 1, 15, 50, 100 points, which represents exposure at this level does not harm the personnel, health effects are reversible and not a threat to one's life, undesirable health effects that are permanent or temporary but have little effect on one's quality of life and life expectancy, and health effects that are usually permanent and can significantly decrease quality of life or life expectancy, respectively. Finally, the equation “RR = C × PrE × PeE × U” was used to calculate the RR.

### Statistical analysis

Results are presented as median and interquartile range (IQR) for continuous data under biased distribution and categorical data. We performed a logarithmic conversion for the concentration of silica-dust before statistical analysis in this study. Exposure time and exposure concentration are also showed as median (range). The permissible concentration time weighted average (PC-TWA) was used to assess if the exposure level of silica-dust and noise exceed the standard in the current study. The ANOVA and LSD-test were used to analyze the differences between different tasks for silica-dust and noise. EpiData 3.1 was used to compile the database and input the data of occupational hygiene survey. All statistical analyses were calculated by SAS version 9.4 (SAS Institute, Cary, NC, USA).

## Results

### Basic information of FMFs in Ningbo

Finally, a total of 193 FMF were included in this study (Table 2). They comprised 14 (7.25%) medium enterprises, 152 (78.75%) small enterprises, and 27 (14.00%) micro enterprises. For process type, there were 103 (53.37%) sand casting foundries and 90 (46.64%) investment casting foundries in the present study. Besides, most of FMFs in Ningbo (131, 67.88%) were located in urban area.

**Table 2:** Basic information of ferrous casting foundries in Ningbo.

**Variables**	**Enterprise size** ^**a**^			**Total**
	**Medium**	**Small**	**Micro**	
**Process type**				
Sand casting	8 (7.77%)	67 (65.05%)	28 (14.00%)	103 (53.37%)
Investment casting	4 (4.65%)	82 (91.10%)	4 (4.65%)	90 (46.64%)
**Region**				
Urban	9 (6.87%)	109 (83.21%)	13 (9.92%)	131 (67.88%)
Rural	5 (8.06%)	43 (69.35%)	14 (22.59%)	62 (32.12%)
Total	14 (7.25%)	152 (78.76%)	27 (13.99%)	193 (100%)

a Enterprise size, the enterprises classification standard was formulated by the National Bureau of Statistics of China.

We observed that silica-dust and noise were the main occupational hazards for the 193 FMFs of Ningbo in the occupational hygiene survey. Sand conditioning, molding and core making, melting, shakeout sand, shot blasting, and cutting and polishing were key locations, which were exposed to the silica-dust and noise.

### Characteristic of occupational hazards

Table 3 shows the key locations and exposure level of silica-dust by different process type. The levels of silica-dust from the majority of location were disqualified both for sand casting foundries and investment casting foundries. For noise, we observed that the levels of noise were qualified from sand conditioning, molding and core making, and melting in sand casting foundries and melting in investment casting foundries. For different locations, the exposure level of silica-dust in shakeout sand was higher than other location (*P* < 0.05) in both sand casting foundries and investment casting foundries. Besides, the exposure level of noise in shakeout sand was also higher than other location (*P* < 0.05).

**Table 3:** Identification of main occupational hazards in ferrous casting foundries.

**Location**	**Risk factor**	**Sand casting**				**Investment casting**			
		**No. of locations**	**Exposure levels [mg/m3 or dB (A)]^a^**	**Length of exposure (median, range) [hours/day]**	**Evaluation by China PC-TWA^b^**	**No. of locations**	**Exposure levels [mg/m3 or dB(A)]**	**Length of exposure (median, range) [hours/day]**	**Evaluation by China PC-TWA**
Sand conditioning	Silca-dust	56	0.48 (0.07–4.33)	8 (4–11)	Disqualified	23	0.35 (0.05–1.79)	6 (2–9)	Disqualified
	Noise	23	81.70 (71.32–98.45)	8 (4–11)	Qualified	14	90.95 (81.70–99.65)	6 (2–9)	Disqualified
Molding and Core Making	Silca-dust	93	1.15 (0.09–4.30)	8 (4–12)	Disqualified	37	0.38 (0.12–2.80)	8 (6.5–11)	Disqualified
	Noise	99	82.90 (74.2–91.6)	8 (4–12)	Qualified	23	82.90 (73.50–90.65)	8 (6.5–11)	Qualified
Melting	Silca-dust	109	0.26 (0–0.90)	8 (4–10)	Qualified	72	0.29 (0.10–1.05)	8 (4–10)	Qualified
	Noise	89	82.70 (74.62–91.38)	8 (4–10)	Qualified	46	85.40 (69.50–91.20)	8 (4–10)	Disqualified
Shakeout sand	Silca-dust	15	1.47 (0.17–5.11)	8 (4–10)	Disqualified	43	0.61 (0.09–2.26)	7.5 (3–11)	Disqualified
	Noise	22	90.75 (78.62–99.78)	8 (4–10)	Disqualified	27	98.60 (85.60–104.35)	7.5 (3–11)	Disqualified
Shot blasting	Silca-dust	39	0.45 (0.03–2.05)	6 (1–12)	Disqualified	34	0.52 (0.09–6.35)	7.5 (3–11)	Disqualified
	Noise	57	88.10 (76.32–101.36)	6 (1–12)	Disqualified	63	89.60 (79.70–99.85)	7.5 (3–11)	Disqualified
Cutting and polishing	Other dust	41	1.50 (0.15–7.40)	7 (4–9)	Disqualified	80	0.43 (0.05–6.35)	7.5 (2–11)	Disqualified
	Noise	60	90.05 (83.62–105.66)	7 (4–9)	Disqualified	75	90.70 (83.40–105.35)	7.5 (2–11)	Disqualified

a The exposure level of silica-dust and other dust is expressed by mg/m^3^, and the exposure level of noise is expressed by dB (A).

b PC-TWA: Permissible concentration-time weighted average.

### Results of occupational risk assessment

In Table 4, we observed that the RRs for silica-dust in the positions of sand conditioning, molding and core making, melting, shakeout sand, and shot blasting were all greater or equal to 400, which represented that workers were exposed to intolerable health risk of silica-dust in the workplace. The RR for 7.32% of No. cutting and polishing was between 200 and 400, which represented very high risk of silica-dust exposure in the workplace. For noise, the RRs in all positions were also greater or equal to 400, which represented that workers were exposed to an intolerable health risk of noise in the workplace.

**Table 4:** Composition of risk ratios (RRs) of different positions for sand casting foundries and investment casting foundries.

**Position**	**OH^a^**	**Concequence**	**Composition of RR (%) for sand casting foundries**						**Composition of RR fro investment casting foundries**					
			**No. of locations**	**≥400**	**200–399**	**70–199**	**20–69**	** < 20**	**No. of locations**	**≥400**	**200–399**	**70–199**	**20–69**	** < 20**
Sand conditioning	Silica-dust	Silicosis	56	100	/	/	/	/	23	100	/	/	/	/
	Noise	Occupational noise deafness	23	100	/	/	/	/	14	100	/	/	/	/
Molding and core making	Silica-dust	Silicosis	93	100	/	/	/	/	37	100	/	/	/	/
	Noise	Occupational noise deafness	99	100	/	/	/	/	23	100	/	/	/	/
Melting	Silica-dust	Silicosis	109	100	/	/	/	/	72	100	/	/	/	/
	Noise	Occupational noise deafness	89	100	/	/	/	/	46	100	/	/	/	/
Shakeout sand	Silica-dust	Silicosis	15	100	/	/	/	/	43	100	/	/	/	/
	Noise	Occupational noise deafness	22	100	/	/	/	/	27	100	/	/	/	/
Shot blasting	Silica-dust	Silicosis	39	100	/	/	/	/	34	100	/	/	/	/
	Noise	Occupational noise deafness	57	100	/	/	/	/	63	100	/	/	/	/
Cutting and polishing	Other dust	Metals and their compounds dust pulmonary disease	41	92.68	7.32	/	/	/	80	95	5	/	/	/
	Noise	Occupational noise deafness	60	100	/	/	/	/	75	100	/	/	/	/

a OH, occupational hazards.

## Discussion

In this study, most of the ferrous metal foundries in Ningbo were small and micro enterprises, which was in accordance with the distribution of previous studies in other cities. This might be related to the overall distribution of enterprises in China. At present, most of FMFs in China are small enterprises, because small enterprises are the main force of development. Besides, it might also be related to the small investment, low cost and flexible operation required by small foundry enterprises.

Silica-dust is the most common occupational hazard in the foundry industry. Silica-dust is one of the most harmful to human health, and the occupational exposure limit (OEL) is the lowest among all dusts. Silicosis caused by silica dust accounts for the largest proportion of pneumoconiosis and is the most harmful. In this study, the dust excess rate of all FMFs, sand casting foundries, and investment casting foundries in Ningbo was 40.61, 37.97%, and 44.11, respectively. However, we found no statistical significant difference between casting process and dust excess rate, which was similar to the previous study (24). The silica dust concentration rate of the main positions was lower than 40%, which was significantly lower than the Shanghai (25) and Jiangsu Province (26). This might be related to the transformation and upgrading of the foundry industry in Ningbo in 2014 (27). Besides, the concentration of silica-dust in sand casting foundries is higher than that in investment casting foundries, which is partly due to the relatively large castings of sand casting technology enterprises, the large amount of sand used, and the poor effectiveness of dust protection facilities (5). The concentration of silica-dust in the shakeout sand of sand casting is higher than that of the sand conditioning and shot blasting. This may be due to the completeness of protective facilities in the shakeout sand is lower than that in the sand conditioning and shot blasting (28). In addition, artificial hammers are usually used to shake the sand and vibrating sand machines are used to shake the sand in the process of shakeout sand, which lead to a serious dust escape, and finally, it is difficult to be effectively captured by local ventilation and dust removal facilities (29).

Silica in foundry dust not only causes silicosis, Gabriella et al. found in a study of two cases of accelerated silicosis that respirable silica could enter the liver and cause granulomas and liver involvement (30). Vihlborg et al. (31) in Sweden In the Iron Foundry Occupational Silica Exposure Risk Study, moderate to high exposure to respirable silica was associated with an increased risk of sarcoidosis and seropositive rheumatoid arthritis. Andjelkovich et al. (32) found that gastric cancer in foundry workers may be associated with respirable silica exposure. In addition to high silica content, foundry dust also contains a certain amount of carcinogenic cadmium, chromium, nickel, etc. and their compounds, as well as other chemically harmful components such as binders and curing agents (33). Studies have shown that these harmful components Causes respiratory and lung inflammation more closely than respirable silica.

In this study, the overall noise exceeding rate of FMFs in Ningbo was 54.47%, among which the noise exceeding rate of sand casting foundries and investment casting foundries were 45.69 and 64.86%, respectively, with the average noise intensity 85.07 and 83.92 dB(A), respectively. The overall noise exceeding rate of sand casting foundries in Ningbo is lower than that in Zhangjiagang City (34), and higher than that of foundry enterprises in Shanghai (25) and Jingjiang City (35). In this study, statistically significant difference was observed between the noise exceeding rate of the sand casting foundries and investment casting foundries, which indicated that FMFs should strengthen noise management, reduce noise pollution, and avoid hearing fatigue or even occupational noise deafness, especially for those adopting the investment casting process. Noise-induced hearing loss is sensory deafness caused by prolonged exposure of the auditory system to a noisy environment (36). Auditory fatigue is an early symptom of noise-induced hearing loss, and hearing can gradually recover after people leave the noisy environment. Prior studies observed that occupational noise exposure is associated with permanent hearing loss (37). The NIH reported that nearly 20 million workers are regularly exposed to noise, of which 50% (10 million) suffer hearing damage of varying severity (38). The WHO estimated that ~16% of disabling hearing impairment results from occupational noise exposure (39). In addition to the damage to the auditory system, noise can also cause damage to the non-auditory system, such as stress, damage to the cardiovascular system, and decline in cognitive and behavioral abilities, so attention should be paid to the impact of noise (40).

The result of occupational health risk evaluation showed that the health risk of silica-dust was the highest level, that is, an intolerable risk (RR ≥400), which was in accordance with the evaluation results of previous studies by using other OHRA models. In addition, we observed that the health risk of noise was also the highest level, which was inconsistent with the result of Gu et al. (40). This may be mainly due to taking different occupational health consequences. However, some occupational health examination results were not available, such as lung and inner ear examination, which limited our further analysis.

In conclusion, there were intolerable risks for silica-dust and noise for FMFs in Ningbo. Exposure to silica-dust and noise in the workplace remains a major concern in the field of occupational health in developing and developed countries, therefore, mature experience in silica-dust and noise control should be performed as soon as possible. Besides, as an important foundry production base in China, it is necessary to supervise enterprises to improve operating environmental conditions, accelerate the reduction of silica-dust and noise exposure risks, and promote the healthy and sustainable development of the foundry industry.

## Data availability statement

The original contributions presented in the study are included in the article/supplementary material, further inquiries can be directed to the corresponding authors.

## Author contributions

DD and AW conceived and coordinated its overall structure. DD, PL, and AW acquired data and performed statistical analyses. DD and PL contributed to the writing and editing of draft. DD worked closely with other authors to align the structure and develop the conclusions. DZ contributed to the overall concept of the draft. DD, PL, XL, GM, DZ, and AW contributed to the editing and revising the draft. All authors interpreted data, revised the manuscript for intellectual content, and approved the final manuscript.
